# The complete chloroplast genome of *Loropetalum chinense* var*. rubrum* yieh (Hamamelidaceae)

**DOI:** 10.1080/23802359.2021.2006094

**Published:** 2021-12-10

**Authors:** Xudong Wang, Heyu Niu, Zhiyuan Huang

**Affiliations:** a School of Architecture, North China University of Water Resources and Electric Power, Zhengzhou, China; b College of Forestry, Henan Agricultural University, Zhengzhou, China

**Keywords:** *Loropetalum chinense* var*. rubrum*, chloroplast genome, Illumina sequencing, phylogenetic tree

## Abstract

*Loropetalum chinense* var*. rubrum* Yieh (*L. chinense*) is an evergreen shrub or small tree of Hamamelidaceae. In this study, the chloroplast genome sequence of *L. chinense* is 159,451 bp in length, consisting of a large single-copy region with 88,166 bp (LSC), a small single-copy region with 18,773 bp (SSC), and two inverted repeat regions with 26,256 bp (IRs). The GC content in the chloroplast genome of *L. chinense* is 38.0%. The chloroplast genome of *L. chinense* contained 125 genes, including 84 protein-coding genes, 37 tRNA genes, and 4 rRNA genes. The phylogenetic tree showed that *L. chinense* was closely related to *L. subcordatum*.

Loropetalum is a genus of Hamamelidaceae, including 4 species and 1 variety, mainly distributed in the subtropical regions of eastern Asia. *L. chinense* var. *rubrum* (*L. chinense*) is a member of the witch hazel family (Hamamelidaceae) and defined as a variety of *L*. *chinense* (Bao 2007). It has been reported that many kinds of unsaturated fatty acids existe in leaves of *L. chinense*, and its leaves could be used as Chinese medicine (Tang 2011). Our complete chloroplast genome data of *L. chinense* can contribute to a better understanding of the evolution of Loropetalum.

The fresh leaves of *L. chinense* were collected in Botanical Garden, Zhengzhou, China (34°44’51”N; 113°32’39”E). The voucher specimen was deposited at the Herbarium of Henan Agricultural University (voucher number:LC-20-0915). The total genomic DNA was extracted from fresh leaves of *L. chinense* using a modified CTAB method (Doyle and Doyle 1987). Sequencing was performed with the Illumina HiSeq2500 Platform (San Diego, CA). The raw reads were generated by Illumina paired-end sequencing after removing adapters. The low-quality sequences of raw reads used Fastp (https://github.com/OpenGene/Fastp) for quality control. Finally, the obtained clean reads were assembled by GetOrganelle pipeline v1.6.3a (https://github.com/Kinggerm/GetOrganelle) with the chloroplast genome of *L. subcordatum* (GenBank accession no. NC_037694.1) as the reference. The genome was automatically annotated by using the CpGAVAS2 pipeline (Shi et al. 2019), and start/stop codons and intron/exon boundaries were adjusted in Geneious 20.2.2 (https://www.geneious.com/).

The chloroplast genome sequence of *L. chinense* was submitted to NCBI, and the accession number was MW346664. The genome sequence of *L. chinense* is 159,451 bp in length, consisting of a large single-copy region with 88,166 bp (LSC), a small single-copy region with 18,773 bp (SSC), and two inverted repeat regions with 26,256 bp (IRs). The GC content in the chloroplast genome of *L. chinense* is 38.0%. The chloroplast genome of *L. chinense* contained 125 genes, including 84 protein-coding genes, 37 tRNA genes, and 4 rRNA genes.

The phylogenetic tree was constructed in RAxML v8.2 (Stamatakis 2006) with 1000 bootstrap replicates. A total of 17 species were used and 2 *Paeonia* species as outgroup (Li et al. 2019). As shown in the phylogenetic tree (Figure 1), the fifteen Hamamelidaceae species were organized into four clusters. The result strongly supported that *L. chinense* was closely related to *L. subcordatum*. This result was similar to the previous phylogenetic trees based on chloroplast genome sequences of Hamamelidaceae (Zhang et al. 2019).

**Figure 1.**
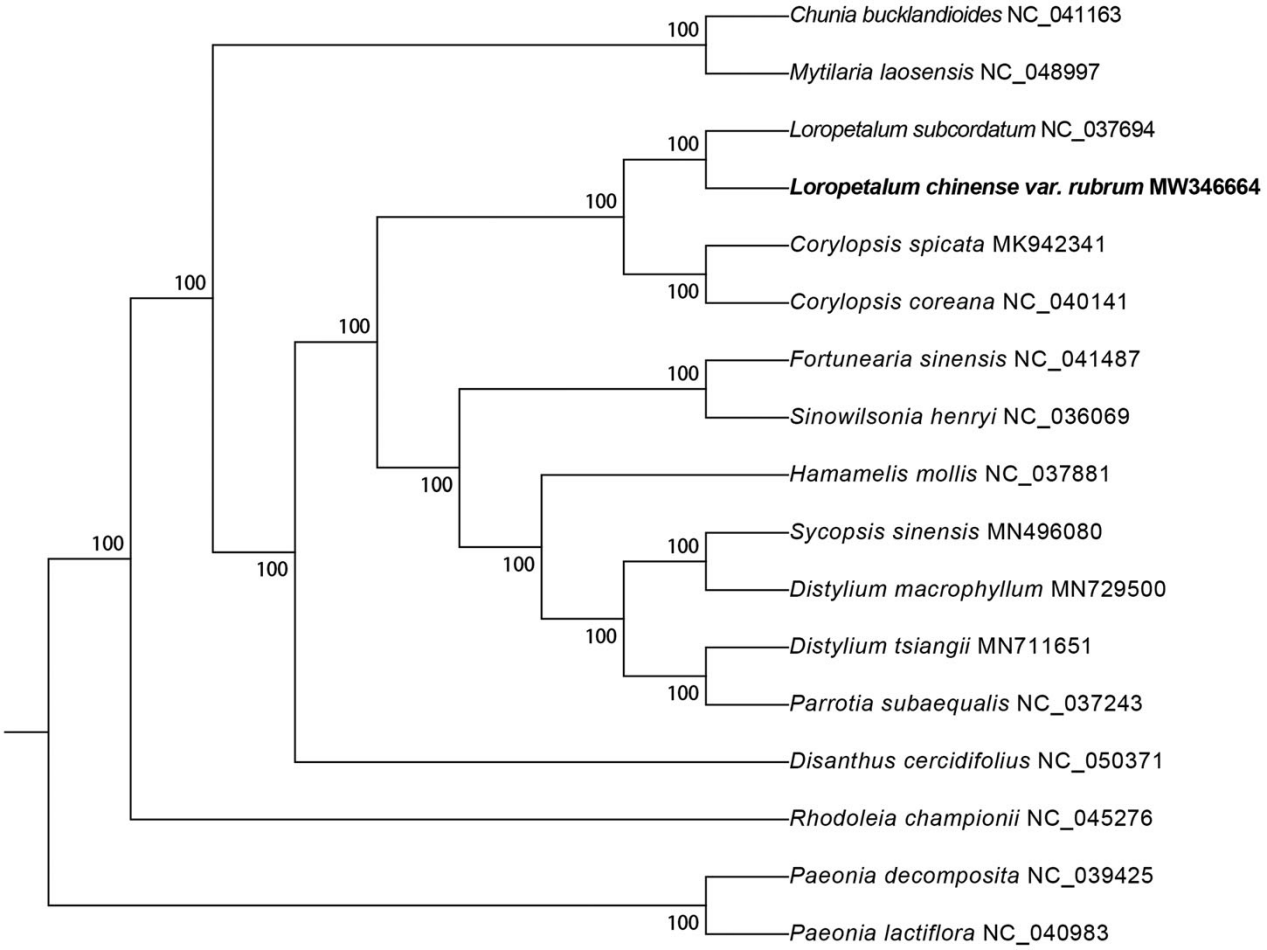
Maximum likelihood (ML) phylogenetic tree inferred from 17 plant chloroplast genomes. Numbers next to the branches are bootstrap support percentages.

## Data Availability

The genome sequence data that support the findings of this study are openly available in GenBank of NCBI at (https://www.ncbi.nlm.nih.gov/) under the accession no. MW346664. The associated BioProject, SRA, and Bio-Sample numbers are PRJNA670186, SRA: SRS7961283, and SAMN17100623 respectively.
